# The Verbal Rating Scale Is Reliable for Assessment of Postoperative Pain in Hip Fracture Patients

**DOI:** 10.1155/2015/676212

**Published:** 2015-05-20

**Authors:** Rune Dueholm Bech, Jens Lauritsen, Ole Ovesen, Søren Overgaard

**Affiliations:** ^1^Orthopaedic Research Unit, Department of Orthopaedic Surgery and Traumatology, Odense University Hospital, 5000 Odense, Denmark; ^2^Institute of Clinical Research, University of Southern Denmark, 5000 Odense, Denmark; ^3^Institute of Public Health, Department of Biostatistics, University of Southern Denmark, 5000 Odense, Denmark

## Abstract

*Background*. Hip fracture patients represent a challenge to pain rating due to the high prevalence of cognitive impairment. *Methods*. Patients prospectively rated pain on the VRS. Furthermore, patients described the changes in pain after raising their leg, with one of five descriptors. Agreement between paired measures on the VRS at rest and by passive straight leg raise with a one-minute interval between ratings at rest and three-minute interval for straight leg raise was expressed by kappa coefficients. Reliability of this assessment of pain using the VRS was compared to the validity of assessing possible change in pain from the selected descriptors. Cognitive status was quantified by the short Orientation-Memory-Concentration Test. *Results*. 110 patients were included. Paired scores with maximum disagreement of one scale point reached 97% at rest and 95% at straight leg raise. Linear weighted kappa coefficients ranged from 0.68 (95% CI = 0.59–0.77) at leg raise to 0.75 (95% CI = 0.65–0.85) at rest. Unweighted kappa coefficients of agreement in recalled pain compared to agreement of paired VRS scores ranged from 0.57 (95% CI = 0.49–0.65) to 0.36 (95% CI = 0.31–0.41). *Interpretation*. The VRS is reliable for assessment of pain after hip fracture. The validity of intermittent questioning about possible change in pain intensity is poor.

## 1. Introduction

Since pain hinders early mobilization, various attempts have been made to improve pain treatment for this category of patients [1, 2]. Adequate alleviation of postoperative pain after hip fracture is crucial for rehabilitation. To evaluate the impact of various approaches to efficient pain treatment a reliable instrument for pain measure is required. A variety of scales are widely used for the assessment of pain, and several studies have been designed to identify pain measurement tools with the most favourable profile for particular groups of patients with regard to specific clinical situations, applicability, and error rate [3, 4]. Hip fracture patients are found primarily among the growing population of the elderly and represent a particular challenge due to their cognitive level that might be impaired, dementia, delirium, or impaired vision or hearing, which makes it difficult to use subtle rating scales [5]. Our data indicates that approximately one-third of the population in the present study suffer from cognitive impairment (Table 4). Difficulties with graphic instruments like the visual analogue scale (VAS) among the elderly have been reported, whereas the Verbal Rating Scale (VRS) has been found to have low error rates [5].

The purpose of our study was to identify an applicable easy-to-handle instrument for formal assessment of pain and we hypothesized that (1) recitation of a five-point VRS for patients would be a reliable tool to assess postoperative pain during hospitalization after hip fracture surgery. The advantages of the VRS for formal postoperative pain assessment in this specific group of hospitalized elderly subjects are that the scale is limited to a few statements, which makes it suitable for reading aloud regardless of lighting conditions and patient's visual power and motor coordination. The VRS is also easy to use in daily clinical practice, since it requires minimal training to use the scale. We further hypothesized that (2) the commonly used practice of evaluating pain by asking the patients to compare actual pain status to previous pain, for example, after administration of analgesics, is not a reliable method in this specific group of patients when comparing to paired formally assessed descriptions of pain on a VRS.

## 2. Material and Methods

### 2.1. Design

We used a prospective cohort design to assess the test-retest reliability of VRS pain measurements. The local ethics committee (Region of Southern Denmark) concluded that approval was not required for this type of questionnaire survey after evaluation of the study protocol. Patients gave their informed consent and the study was reported to the Danish Data Protection Agency (Copenhagen, Denmark).

### 2.2. Setting and Subjects

**Figure 1:**
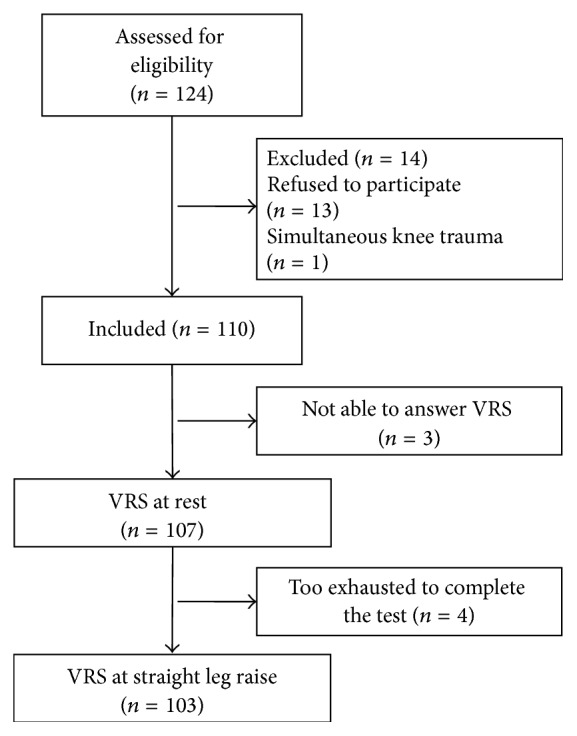
Flowchart of patients. 107 paired measures were assessed at rest. 103 patients completed paired measures at straight leg raise.

During a six-month period, patients who have had osteosynthesis of a hip fracture in three orthopaedic departments in the Region of Southern Denmark were interviewed 0 to 17 days (mean = 3.55, 95% CI = 3,03–4,07) after surgery in hospital (Figure 1). Data were collected by three trained research assistants on two specific weekdays during the study period. All patients met the following inclusion criteria: They were Danish speaking, hospitalized after surgical repair of a unilateral hip fracture caused by a low-energy trauma, classified as femoral neck fractures, pertrochanteric fractures, or subtrochanteric fractures (AO/OTA type 31-A1 to 31-A3 or 31-B1 to 31-B3). Patients who could not participate due to medical conditions or severe hearing loss were excluded.

### 2.3. Measures

Variables of cognitive status were assessed using the Danish version of the inverted short Orientation-Memory-Concentration Test (sOMC) [6]. The sOMC has a possible maximum of 28 points and a score less than 18 points indicates significant cognitive impairment [7, 8]. A score below eight points indicates severe cognitive impairment [9]. No distinction was made between subjects who were cognitive impaired by delirium and those who had a dementing illness. We used a five-point Verbal Rating Scale (VRS) with the words “no pain,” “slight pain,” “moderate pain,” “severe pain,” and “unbearable pain” (in Danish, “ingen smerte,” “let smerte,” “moderat smerte,” “svær smerte,” and “uudholdelig smerte”). Assessment of pain was done by asking bed-ridden patients to rate their present intensity of pain in the hip/thigh at rest, by indicating which of the five words read aloud gave the best description of their present pain. The possible answers were read out again if the patient asked for a repetition, or if the research assistant considered that the patient needed to get the answers possibilities repeated. One minute later patients were again asked to rate their present intensity of pain without reference to the first measurement. The one-minute time interval was chosen under the assumption that most pain at rest would not change within a one-minute period. Just after this procedure the investigator elevated the patient's leg to induce straight leg raise to twenty degrees of hip flexion and the VRS was repeated before the leg was lowered to rest position again. Three minutes later patients were once more asked to rate their present intensity of pain by passive straight leg raise to twenty degrees of hip flexion without reference to the first measurement. We expected straight leg raise to intensify pain which could influence the distribution of VRS scores. We chose the three-minute time interval under the assumption that any aggravation of pain from the elevation of the leg would normalize during this period.

**Figure 2:**
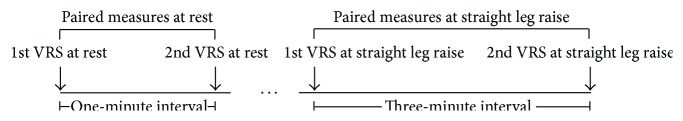
Diagram of pain rating.

Our scorings resulted in one set of paired measurements at rest and one set of paired measurements at straight leg raise (Figure 2). After completing the second VRS score at rest, patients were also asked to compare current pain with their pain at the previous assessment of pain at the first VRS at rest, and after completing the second VRS scores at straight leg raise patients were again asked to compare current pain with their pain at the previous assessment of pain at the first VRS at straight leg raise. The subjects had to rate the possible changes in pain by one of five categorical descriptors: “much less pain,” “a little less pain,” “the same pain,” “a little more pain,” or “much more pain” (in Danish, “meget mindre smerte,” “lidt mindre smerte,” “samme smerte,” “lidt mere smerte,” or “meget mere smerte”), which were read aloud.

### 2.4. Data Analysis

Data were entered into EpiData 3.1 (EpiData Association, Odense, Denmark) and double-checked for entry errors before export to Stata 10.1 (StataCorp LP, Texas, USA) for statistical analysis. To reflect the agreement between the paired measurements we used the linear weighted kappa coefficient (*κ*_*w*_) [11] which allows minor disagreement between ratings but attaches greater emphasis to large differences between ratings. Recalled pain assessed using the five categorical descriptors was converted into three categories: “less pain,” “the same pain,” and “more pain.” These three categories were compared to delta values of the paired VRS scores which were also converted into three categories: “decrease in VRS,” “unchanged VRS,” and “increase in VRS.” Because these data were collapsed into compound categories the degree of agreement had to be evaluated by unweighted kappa (*κ*). The kappa coefficient represents the proportion of agreement greater than expected by chance. For intermediate kappa values between 0 and 1, Landis and Koch [12] have proposed the following interpretation: below 0.0 = poor, 0.00–0.20 = slight, 0.21–0.40 = fair, 0.41–0.60 = moderate, 0.61–0.80 = substantial, and 0.81–1.00 = almost perfect.

## 3. Results

Tables 1 and 2 show the distribution of the paired scores.

### 3.1. Demographic Characteristics of Subjects

A total of 110 patients were interviewed once between postoperative days 0 and 17 (mean = 3.55, 95% CI = 3.03–4.07) (Figure 1). The average age was 80 years (range 46 to 99 years). 80 were females (73%).  Table 3 shows the distribution of fractures and Table 4 shows the distribution of sOMC scores in the study population after stratification according to sOMC score. Approximately one-third had a sOMC score indicating cognitive impairment.

### 3.2. Ability to Reply to the VRS Statements

A majority of the patients were able to rate their pain using the VRS (Figure 1). Only three patients were not able to reply to the VRS and they all had a sOMC score of zero. We assessed 107 (97%) paired measures one minute apart at rest and 103 (94%) paired measures three minutes apart at passive hip flexion. A total of 103 (94%) patients were able to complete the paired measures of pain at rest and pain at hip flexion, respectively.

### 3.3. Reliability

**Figure 3:**
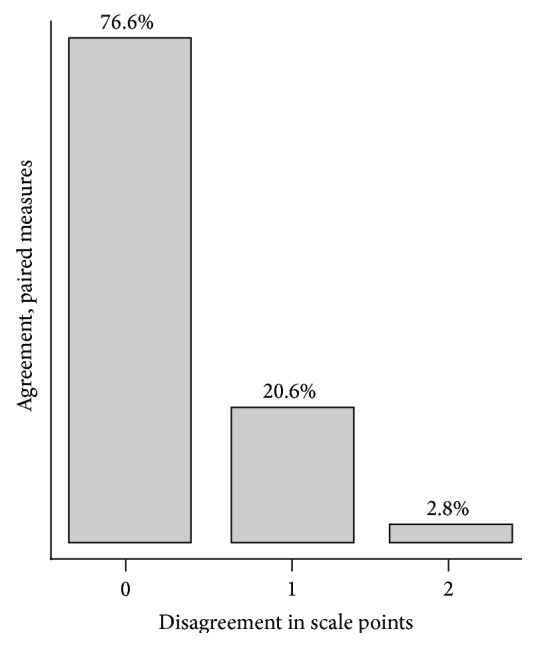
VRS at rest. The bars and percentages show disagreement of 0, 1, and 2 scale points between the paired scores. Maximum disagreement was 2 scale points.

**Figure 4:**
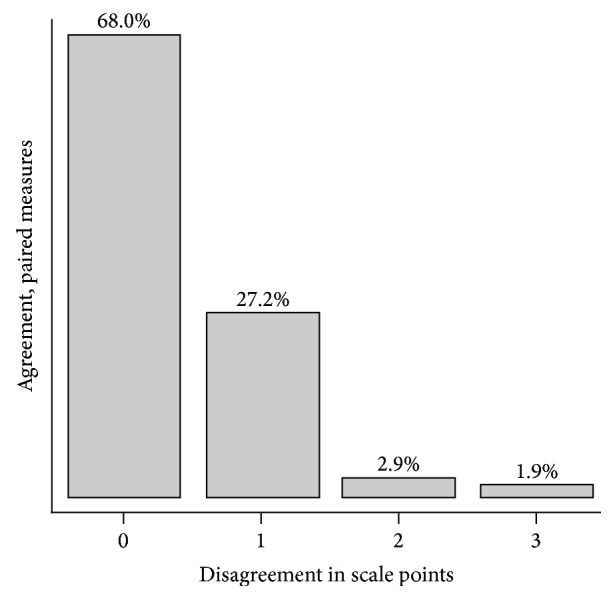
VRS at straight leg raise. The bars and percentages show disagreement of 0–3 scale points between the paired scores. Maximum disagreement was 3 scale points.

Figures 3 and 4 show the relationship between the VRS scores assessed at rest and the relationship between the VRS scores at straight leg raise. At rest perfect agreement is seen in 77% of the paired scores while 97% of the paired scores have a maximum disagreement of one scale point. At straight leg raise the perfect agreement between the paired scores is 68% compared to 95% with a maximum disagreement of one scale point. Tables 5 and 6 show the degree of agreement between the paired scores quantified by *κ*_*w*_. When comparing agreement of the paired scores obtained from the whole population (sOMC 0–28) at rest and by straight leg raise with the previously mentioned interpretation proposed by Landis and Koch [12], the *κ*_*w*_-measures of 0.75 and 0.68 reach “substantial” agreement. The lowest agreement is observed by straight leg raise among the subjects stratified with most severe cognitive impairment (sOMC 0–7). The *κ*_*w*_ of 0.44 is interpretable as “moderate agreement.” Interestingly, the same group of severely impaired patients actually shows the best agreement at rest reaching a *κ*_*w*_ of 0.83 which is interpretable as “almost perfect.”

Tables 7 and 8 show the degree of agreement between changes in the paired VRS scores and changes in recalled pain quantified by kappa. The agreement between recalled pain and delta values of VRS scores obtained by formal assessment of pain is poor.

## 4. Discussion

To our knowledge this is the first study to investigate retest reliability of a five-point VRS for assessment of postoperative pain during admission to hospital after hip fracture surgery. Since the obtained kappa-statistic measures of test-retest agreement of 0.68–0.75 at rest and by induced pain by straight leg raise can be interpreted as “substantial” [12], our primary hypothesis that recitation of the five-point VRS is reliable for evaluation of postoperative pain during hospitalization after hip fracture surgery is supported.

Also the second hypothesis that evaluation of pain by simply asking the patients if pain has changed, for example, after administration of analgesics, does not consistently lead to correct answers in this specific group of patients compared to formal assessment of pain assisted by the VRS is supported since kappa-statistic measures of test-retest agreement in the whole population only reached 0.57 after one minute and 0.36 after 3 minutes.

Our findings also indicate that cognitive intact and cognitive impaired patients from this population are able to report pain reliably using the VRS, which supports findings in other patient populations and in studies using other versions of the scale [3, 13].

Several factors such as poor short-term memory, pain, medication, constant light conditions, and lack of familiar routines, which are necessary to sense time and to connect specific events, may possibly explain the inability to compare pain as experienced at different times. Nevertheless this fact emphasizes the importance of implementation of formal pain measurements tools as the VRS.

In previous studies the reliability of slightly different versions of the VRS has been assessed [5, 14], and the specific five-point version used in this study appears to be applicable in the elderly [15, 16]. To our knowledge, we are the first to evaluate recitation of a five-point version of the scale for assessment of postoperative pain after hip fracture surgery.

Hip fracture is a common event in the geriatric population, and it is associated with significant pain and loss of function. Unfortunately, undertreatment of postoperative pain remains a persistent problem. Previous studies have found that hip fracture patients generally suffer from substantial postoperative pain and that age and cognitive impairment strongly influence the administered amount of analgesics in a negative direction [13, 14], even though no evidence indicates that cognitive impairment or age changes the perception of pain [17, 18]. The VRS is a simple tool limited to a few statements, and it appears to be the most usable tool for pain assessment in cognitively impaired subjects [3, 5, 15, 16].

We chose the sOMC to evaluate cognitive status because this test does not require the patient to perform drawings, which requires a table surface and possible the use of glasses and sufficient light source as, for example, MMSE [19]. In addition, our staff is experienced in the use of sOMC, which is free to use.

The VRS is among the earliest tools for formal pain measurement, and several versions of the scale exist [20, 21]. The lack of consistency in items and descriptors makes comparison of data from different studies difficult. Also the fact that international publications are mainly printed in English results in that the VRS used in, for example, a Danish study, is presented in an English translation that might not exactly reflect the items used, if specified at all. Based on the assumption that too few items would affect the ability to register minor changes in pain and that too many items might cause overlap in the meaning of the items, we chose a five-point version of the scale. “Mild” to one person may mean “slight” pain to another if too many ranking points are chosen. We considered that there was no overlap in the meaning of the selected words, and we considered that the included words did not require any particular educational level or rich vocabulary, for example, the McGill Pain Questionnaire [20], “discomforting, distressing, horrible, and excruciating.”

The VRS has been criticised due to the few response categories that may not be suitable to register small changes in pain. However, even though an extension of the number of response categories could potentially increase the sensitivity of any scale, this would not necessarily make the scale more valid since small changes are not necessarily clinically meaningful or easier to interpret. However, there are statistical limitations connected to analysis of data generated from the VRS. Even though the scale categories are ranked according to severity of pain, the scale does not measure an exact relative difference between the descriptors which makes the scale ordinal. Thus, nonparametric statistics has to be used resulting in limited sophistication of statistical evaluation. Another point of criticism about the VRS is that the composition of discrete categories, of which the respondent must choose only one, induces an element of forced choice [21]. We agree with this assumption, but our data suggest that, in this specific category of frail patients which contains a considerable prevalence of cognitive impairment, guidance by means of a limited number of predefined response options actually brings about reliable assessments of pain.

A weakness in our study is the lack of correlation to an objective “gold standard” or a corresponding scale to evaluate concurrent validity, but we actually considered the VRS to be so simple that no other scale would be directly comparable. It is also debatable if the paired answers are affected by the recall of the previous statements. We cannot exclude this influence from previous answers, but since the subjects had no detailed knowledge of the analysis of paired statements we consider that they were in fact focused on describing their actual pain rather than trying to repeat the previous statement, which could be different from their actual pain. Nevertheless we acknowledge that the possibility exists.

Other papers report similarly that the VRS appears to be applicable in the elderly with a cognitive dysfunction [22–27] and one might ask if it is necessary to show that VRS is reliable to measure pain in elderly undergoing just this specific surgical procedure. However, hip fractures are a common result of fall among the older age groups of the population. In Denmark, approximately 10.000 patients with a mean age of 80 years are admitted to hospital per year with a hip fracture [28]. The significant number makes these fractures a major cause of morbidity and treatment of postoperative pain is important to facilitate early mobilisation and rehabilitation. Our desire to offer this large group of patients the best possible treatment is the direct cause of our study. No therapy should be initiated without a specific end-point. Formal assessment of pain helps us to communicate, to verify, and to document the effect of analgesic therapy, which makes the introduction of readily applicable tools for pain rating into the daily routines of postoperative care essential. For successful introduction of such new tools, they must be easy to use and they must provide reliable information. The VRS is simple and easy to use, and based on the data from this study it seems to be applicable for assessment of postoperative pain in patients admitted to hospital after hip fracture surgery.

## Figures and Tables

**Table 1:** Paired VRS scores at rest.

	2nd VRS					Total
	No pain	Slight pain	Moderate pain	Severe pain	Unbearable pain	
1st VRS						
No pain	36	3	1	0	0	40
Slight pain	4	20	4	0	0	28
Moderate pain	2	6	23	0	0	31
Severe pain	0	0	5	2	0	7
Unbearable pain	0	0	0	0	1	1

Total	42	29	33	2	1	107

**Table 2:** Paired VRS scores at straight leg raise.

	2nd VRS					Total
	No pain	Slight pain	Moderate pain	Severe pain	Unbearable pain	
1st VRS						
No pain	30	7	1	1	0	39
Slight pain	5	20	6	0	0	31
Moderate pain	1	3	13	3	0	20
Severe pain	0	0	1	5	2	8
Unbearable pain	0	1	1	1	2	5

Total	36	31	22	10	4	103

**Table 3:** Fractures.

Fracture	Frequency	Percent
Femoral neck fractures	58	53
Pertrochanteric	42	38
Subtrochanteric	10	9

Total	110	100

**Table 4:** Distribution of sOMC scores.

sOMC score^*∗*^	Frequency	Percent
0–7 (severe cognitive impairment)	14	13
8–17 (significant cognitive impairment)	25	23
18–28 (no cognitive impairment)	71	64

Total	110	100

^*∗*^Originally the stratification of sOMC scores is based on error scores [10]. To facilitate understanding the intervals are adjusted to the scoring format used [9].

**Table 5:** Agreement between paired VRS scores at rest, quantified by weighted kappa (*κ*_*w*_).

	Paired VRS	Agreement %	Expected agreement %	*κ*_*w*_	(95% CI)
All patients (sOMC 0–28)	VRS at rest	94%	74%	0.75	(0.65–0.85)
sOMC 0–7	VRS at rest	94%	65%	0.83	(0.50–1.15)
sOMC 8–17	VRS at rest	92%	67%	0.76	(0.53–0.99)
sOMC 18–28	VRS at rest	91%	66%	0.72	(0.60–0.85)

**Table 6:** Agreement between paired VRS scores at straight leg raise, quantified by weighted kappa (*κ*_*w*_).

	Paired VRS	Agreement %	Expected agreement %	*κ*_*w*_	(95% CI)
All patients (sOMC 0–28)	VRS at straight leg raise	90%	70%	0.68	(0.59–0.77)
sOMC 0–7	VRS at straight leg raise	78%	61%	0.44	(0.28–0.60)
sOMC 8–17	VRS at straight leg raise	93%	70%	0.75	(0.55–0.95)
sOMC 18–28	VRS at straight leg raise	91%	71%	0.68	(0.58–0.79)

**Table 7:** Reliability of recalled pain from rest to straight leg raise. The five possible descriptors of recalled pain are converted into three categories: “less pain,” “the same pain,” and “more pain.” The table shows the change in pain from 2nd VRS at rest to 1st VRS by straight passive leg raise.

	Change in pain	Agreement %	Expected agreement %	*κ*	(95% CI)
All patients (sOMC 0–28)	2nd VRS at rest to 1st VRS by straight leg raise	73%	36%	0.57	(0.49–0.65)
sOMC 0–7	2nd VRS at rest to 1st VRS by straight leg raise	56%	38%	0.28	(0.16–0.40)
sOMC 8–17	2nd VRS at rest to 1st VRS by straight leg raise	70%	36%	0.52	(0.37–0.68)
sOMC 18–28	2nd VRS at rest to 1st VRS by straight leg raise	76%	36%	0.62	(0.52–0.73)

The consecutive pain ratings are outlined in Figure 2.

**Table 8:** Reliability of recalled change in pain by straight leg raise at 3-minute interval. The five possible descriptors of recalled pain are converted into three categories: “less pain,” “the same pain,” and “more pain.”

	Change in pain	Agreement %	Expected agreement %	*κ*	(95% CI)
All patients (sOMC 0–28)	1st VRS by straight leg raise to 2nd VRS by straight leg raise	68%	50%	0.36	(0.31–0.41)
sOMC 0–7	1st VRS by straight leg raise to 2nd VRS by straight leg raise	43%	37%	0.10	(0.05–0.14)
sOMC 8–17	1st VRS by straight leg raise to 2nd VRS by straight leg raise	70%	54%	0.35	(0.23–0.47)
sOMC 18–28	1st VRS by straight leg raise to 2nd VRS by straight leg raise	70%	52%	0.39	(0.32–0.46)

The consecutive pain ratings are outlined in Figure 2.
